# Oxidative Stress-Induced Neurodegeneration and Antioxidative Strategies: Current Stage and Future Perspectives

**DOI:** 10.3390/antiox12091762

**Published:** 2023-09-14

**Authors:** Ana-Maria Buga, Carmen-Nicoleta Oancea

**Affiliations:** Department of Biochemistry, University of Medicine and Pharmacy of Craiova, 200349 Craiova, Romania; ana.buga@umfcv.ro

Neurodegenerative diseases (NDs) are the leading cause of neurological disorders, constituting a public health problem with an exponentially growing incidence rate [1]. By 2024, NDs are estimated to become the second-leading cause of mortality in the world [2]. The ageing population are at increased risk of NDs and stroke. Among the tissues, the brain is more susceptible to neurodegeneration. This process increases with age, and many researchers have asked whether the neurodegeneration process is a hallmark of ageing. However, major NDs affect people over 65 years old. Alzheimer’s disease (AD) and Parkinson’s disease (PD) are the leading NDs. According to the World Health Organization (WHO), Alzheimer’s disease (AD) is the most common form of dementia worldwide and affects more than 50 million people over the age of 65. AD is misdiagnosed due to the fact that many people with mild cognitive impairments (MCIs) progress to AD [3]. In addition, ischemic stroke is not currently recognized as a neurodegenerative disorder, but a chronic neurodegenerative process appears after the initial phase of ischemic stroke in brain areas far away from the lesion [4,5]. Preclinical and clinical data show that post-stroke brains undergo long-term structural changes that lead to brain atrophy [5,6]. These structural changes represent the hallmark of neurodegenerative diseases, but it remains to be established whether there is a direct link between ischemic stroke survivors and an increased risk of AD. Neurodegeneration is not only an old-age health problem; it also affects young adults. Diseases such as multiple sclerosis (MS) and amyotrophic lateral sclerosis (ALS) are accompanied by neurodegeneration. MS and ALS affect the young, active population with a cumulative incidence of 3 million people worldwide (2.8 million for MS and 200.000 for ALS) [7]. However, brain tissue is not the only part of the human body that can be damaged; age-related macular degeneration (AMD) and glaucoma are characterized by neurodegeneration and permanent damage to the eyes [8]. There are currently no therapeutic interventions available to cure these disorders. Despite research efforts to cure NDs, the majority of promising interventions have failed in clinical trials. A common feature of many NDs is the enhancement of oxidative stress and inflammation that trigger mitochondrial disfunction and cellular death. In NDs, OS promotes the propagation of pathophysiological processes that determine the progression of most neurodegenerative states.

At the end of 2022, PubMed found that over 400,000 articles had been published regarding neurodegenerative diseases, and about 18,000 of these included references to oxidative stress along with mechanisms interconnected with NDs [9]. In this Special Issue (SI) of *Antioxidants*, entitled “Novel Therapies of Oxidative-Stress-Induced Age-Related Neurodegenerative Diseases”, we highlighted the latest findings regarding neurodegeneration, antioxidants, nutrigenetics, nutraceuticals, or mitochondrial dysfunction. Since there is wide diversity in the ways neuroprotection can be obtained and in the ways neurodegenerative conditions can be treated, achieving neuroprotection and treating NDs, according to statistics, will become increasingly easy. This SI is well balanced and comprises five critical reviews and four original articles. These articles aim to review the recent findings in the ND field and highlight the gaps in knowledge and future research directions. The reviews cover important directions such as (i) the identification of new potential biomarkers for age-related ND disease progression that can be modulated for therapeutic purposes [10]; (ii) the development of a new preclinical model for PD to test new potential drugs, with an increased potential to limit disease progression or cure it [11]; (iii) the role of nutraceuticals and physical exercise in controlling oxidative balance and improving functional outcomes in NDs [12,13]; (iv) mitochondrial function and oxidative stress in relation to successful cellular therapy for stroke [14].

Juan Segura-Aguilar and his colleagues studied a KEAP1/NRF2 transcriptional activation preclinical model for PD, because no current treatments for PD are guaranteed to work, and no one has studied the effect of antioxidants on idiopathic PD [11]. This model will be useful for the development of new potential antioxidant therapies [11]. This is an interesting study that led to the discovery of several phytochemical activators of KEAP1 (Kelch-like ECH-associated protein (1)/NRF2 (nuclear factor erythroid-derived 2-like (2), which can inhibit or decrease aminochrome-induced neurotoxicity [11]. In addition, Llido and colleagues provided evidence in relation to serum bilirubin levels as a predictor for the evolution of neurodegenerative diseases [10]. Bilirubin is a potent antioxidant molecule, and decreasing its concentration can predict disease progression.

The therapeutic potential of nutraceuticals, such as catechins, to limit the neurodegenerative process in glaucoma was reviewed by Tsz Kin Ng and colleagues, and the potential clinical benefits were emphasized [12]. They studied the pharmacokinetic aspects and therapeutic properties of catechins that underlie the possibility of using catechins, phenolic compounds particularly found in green tea, as adjuvants in clinical treatments of neurodegenerative diseases due to their antioxidant and anti-inflammatory properties [12]. New approaches to oxidative-stress-related to physical activity and nutraceuticals in normal ageing and neurodegenerative ageing were highlighted by Manuela Violeta Bacanoiu and colleagues. Physical exercise and nutraceuticals display a protective antioxidant effect by decreasing free radicals and proinflammatory markers [13].

Also, four original articles were published, with the aim of better understanding the role of oxidative stress in NDs and identifying new potential therapeutic interventions in order to restore redox balance. One study published in this SI by [15] and colleagues evaluated the neuroprotective mechanisms exerted by black pepper extract. Black pepper, native to South Africa and also called “Black Gold”, is commonly used to treat colds, neuropathic pain, and respiratory diseases [15]. It has been shown to have anti-AChE and anti-amyloid activity and can restore antioxidant enzyme levels. It has also been shown to reduce ROS production and maintain mitochondrial membrane integrity in neuroblastoma cell cultures. ROS production is considered an important mediator of oxidative stress, neuroinflammation, and cell death [15]. Piperine, the main alkaloid of black pepper, has demonstrated anti-AChE and anti-amyloid activity. The study also showed competitive acetylcholine esterase (AchE) inhibition with promising cytotoxicity (IC50) values, the significant inhibition of Aβ fibrilization, and strong anti-glycation activity with the prevention of advanced glycation end-product (AGE) formation. Black pepper, through its multitarget neuroprotective mechanism, may represent a first step in the development of neurodegenerative disease therapies [15].

Another nutraceutical compound with antioxidant activity that was demonstrated in an article published in this Special Issue by Lee and colleagues is *Dipterocarpus tuberculatus Roxb*. Through its seven bioactive components, the methanolic extract of Dipterocarpus (MED) was able to strongly capture free radicals of 2,2-diphenyl-1-picrylhydrazyl (DPPH) and 2,2′-azono-bis-3-ethylbenzthiazoline-6-sulphonate (ABTS) [16]. MED treatment suppresses the increases in nitric oxide (NO) concentrations and reactive intracellular oxygen species (ROS), with a significant increase in superoxide dismutase (SOD) enzyme activity and recovery of antioxidant capacity. Protective effects have been demonstrated in retinal degeneration such as improving retinal thickness, the inner nuclear layer (INL), the photoreceptor layer (PL), and the outer nuclear layer (ONL) in Balb/c mice [16]. The well-known vitamin, vitamin C, with proven antioxidant properties, was studied by Nery Jara and colleagues in terms of the worldwide deficiency [17]. The study highlighted significantly higher prevalences of deficiency in developing countries with negative consequences in human neurogenesis.

The complexity of the theme of this SI also brought to light the study conducted by Franziska T. Wunsch and colleagues, who studied defects in the glutathione system in an animal model of ALS [18]. The study, with an impressive bibliography, revealed that the reduced amount of glutathione obtained in the cervical spinal cord of wobbler mice is due to a decrease in glutathione synthesis due to a decrease in the expression of the speed-limiting enzyme glutamyl-cysteinyl-ligase. For the first time, evidence has been provided for impaired glutathione metabolism in ALS in wobbler mice, not limited to motor areas of the CNS, with the need to study whether reduced glutathione is a causative factor or consequence of neurodegeneration into ALS, but with the opening of a possible targeted therapy for ALS by specifically improving glutathione synthesis [18].

The significant importance of functional mitochondria in reducing oxidative damage by restoring mitochondrial integrity suggests a revolutionary effect in treating stroke with reduced neuroinflammation and reperfusion injury, which has been studied and published by Molly Monsour and colleagues [14]. With a better understanding of how important functional mitochondria are in recovering from a stroke, big improvements can be made in promoting mitochondrial transfer and making stem cells more useful for therapy.

This Special Issue, “Novel Therapies of Oxidative-Stress-Induced Age-Related Neurodegenerative Diseases,” published nine papers, including four original research papers and five review articles, all of which addressed different aspects of neurodegenerative diseases, innovative therapies in NDs, and correlations of oxidative stress in their induction.

We would like to express our full appreciation to the authors who contributed papers to this Special Issue. The remarkable quality of these studies will certainly add new content and generate new openings in multidisciplinary research into the role of oxidative stress and the complex mechanisms underlying potential therapeutic benefits in neurodegenerative diseases.
